# Long-Range Vapor-Mediated Interactions between Adjacent Droplets

**DOI:** 10.1021/acs.langmuir.4c04255

**Published:** 2025-02-05

**Authors:** Hongyu Zhao, Daniel Orejon, Khellil Sefiane, Martin E. R. Shanahan

**Affiliations:** † Institute for Multiscale Thermofluids, School of Engineering, The University of Edinburgh, King’s Building’s, Mayfield Road, Edinburgh EH9 3FD, U.K.; ‡ Arts et Metiers Paris Tech, University of Bordeaux, I2M, UMR 5295, F-33400 Talence, France

## Abstract

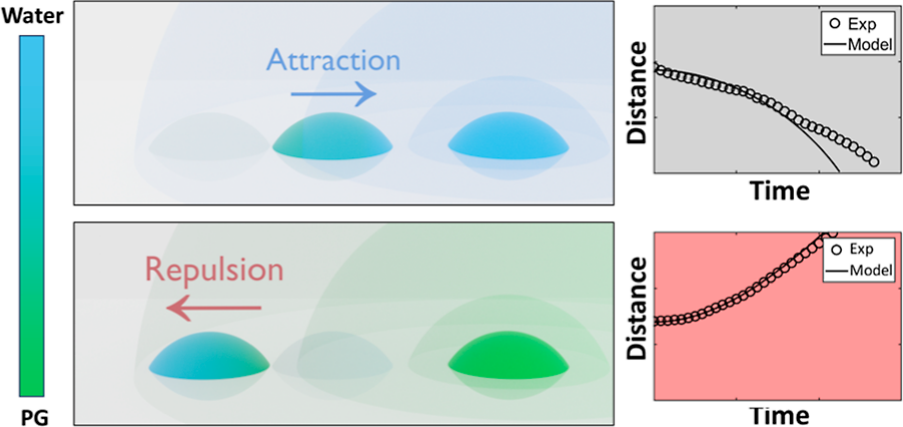

Droplet motion can occur due to interaction with the surrounding vapor phase. We examined experimentally the motion of two adjacent droplets, either pure liquid or a binary mixture, without direct contact. A droplet is repelled or attracted by the (pinned) adjacent droplet, which acts as a vapor source, depending on its initial concentration as well as the composition in the vapor, even for a pure liquid. The observation is explained by a theoretical model that combines evaporation and adsorption processes, which unifies the mechanism for both directions of motion (attraction and repulsion) and, more importantly, for both binary mixtures and pure liquid droplets. Good agreement is achieved between the theoretical model and the experimental observations. A critical concentration is proposed to determine the transition between attractive and repulsive motion, this being a criterion to predict droplet motion.

## Introduction

Microscale droplet migration has been investigated using different activation or functional approaches, which include passive ones such as chemical^1,2^ and/or structural gradients^3,4^ on the surface, or active ones such as electrowetting,^5^ vibration,^6^ light,^7^ thermal gradient,^8^ etc. While physical or chemical gradients require specifically engineered complex surfaces and fabrication steps, external forces, which can be costly or time-consuming, limiting the potential application of droplet motion, are required for other active approaches.

Compared to the above introduced approaches, directional motion of a liquid droplet can be simply induced by the vapor to which the droplet is exposed.^9−16^ Bangham and Saweris^9^ were the first to study the motion of pure droplets induced by the vapor of another component in 1938. Their conclusion was that a droplet with a lower surface tension moves toward the vapor of another liquid with a higher surface tension, resulting in attraction. In contrast, a droplet with a higher surface tension moves away from the vapor, leading to repulsion. In their explanation, the vapor is adsorbed onto a thin precursor film around the droplet influencing the local contact angle. Later works suggested that for vapor-driven droplet motion, the vapor could be generated not only from a capillary^9,10^ but also from another adjacent droplet of different composition,^11−16^ while the droplet could be either a pure liquid droplet,^9,11−13,15,16^ or a binary mixture droplet.^10,11,14,16^ Nonetheless the proposition of a general mechanism that can account for both the attraction and repulsion for pure and binary droplets is lacking.

To understand the motion of droplets driven by vapor gradients, different explanations and models have been proposed in different situations. A conventional explanation for such motion of a pure liquid droplet is that the vapor of another component is adsorbed onto the liquid–vapor interface of the droplet or onto its precursor film, which alters the concentration and thus its surface tension locally along the interface and near the triple phase contact line. Therefore, a gradient of vapor leads to a gradient of surface tension, which in turn leads to the directional motion of the pure droplet.^9,12,13^ The occurrence of adsorption to explain such changes in surface tension, empowering the droplet motion, can be adequate for pure liquid droplets. Besides, Barrio-Zhang et al.^29^ looked into the motion of pure droplet induced by the gradient of its own vapor. However, for the cases of binary mixture droplets, the situation becomes more complex. The variation of the evaporation rate of different components and that of the same component subjected to different vapor concentrations can also lead to a concentration gradient along the droplet liquid–vapor interface.^10,14^ Some researchers argued that this was the main mechanism driving the binary droplet.

More recently, Cira et al.^14^ reported attractive and repulsive motion of binary mixture (water and propylene glycol, PG) droplet pairs and the dependence on their concentrations. The model they proposed, based on a mechanism involving the surface tension gradient arising from the spatial water evaporation rate variation of the droplet subjected to a water vapor gradient, could not explain the repulsive motion of the droplet or the motion in the presence of pure component droplets. Hence, thereafter, Jiang et al.^11^ proposed a model to explain the repulsive motion of a sessile droplet (water-PG mixture or pure water) away from an isopropyl alcohol pendant droplet from a capillary tube sitting above the substrate. They assumed that the surface tension of the precursor film extending away from the droplet always adjusts to the surrounding phase. However, this assumption leads to the same driving force for a given vapor profile regardless of the different initial concentrations of the moving droplet, which has proven to be controversial in the light of the experimental observation of both attractive and repulsive motions reported earlier by Cira et al.^14^ In the same line of research, Man and Doi^17^ theoretically analyzed the effects of nonuniform evaporation rate and Marangoni effect separately to explain the motion of two pure droplets of the same liquid. In their explanation, the nonuniform evaporation rate does not contribute to the surface tension gradient as in the explanation of Cira et al.^14^ Instead, it induces a capillary force, which could drive the droplet, even without the presence of a spatial surface tension variation. The latter mechanism was demonstrated by the theoretical work of Wen et al.^15^ supporting their observation of the attraction of identical pure droplets. However, a fixed surface tension gradient is given in the model of Man and Doi, which is not related to either the vapor gradient or the initial concentration of the droplet and therefore is unable to explain the moving direction alternation due to the change in initial concentration of the free-moving droplet given a fixed pinned droplet concentration.^14^ Researchers have also looked into the internal convection of the droplet influenced by the vapor from an adjacent droplet,^18−20^ which states that strong internal convection can be induced by the solutal Marangoni effect. However, without the tuning of vapor profile and droplet concentration, the relation between the moving direction and the vapor profile is unclear.

To summarize, different experimental observations and theoretical models have been proposed to date in the literature exploring the interaction between two adjacent binary mixtures or pure liquid droplets. However, no quantitative theory has been successfully developed to fully explain the mechanism behind the vapor-driven motion, or to successfully capture the observations including both attractive and repulsing motion for both pure and binary mixture droplets.

In this work, we argue that both the evaporation process and the adsorption process take place during the interaction of two adjacent droplets, the magnitudes of which depend on the concentrations of the two droplets and the (local) relative humidity. The overall effect of mass transfer plays a significant role in altering the concentration within the liquid at the liquid–vapor interface of the free-moving droplet through the interaction with the surrounding vapor, whether the droplet is binary or pure. Therefore, variations in concentration across space result in variations in surface tension, generating a net force that dictates whether the interactions are repulsive or attractive. A complete phase diagram accessing pure water and PG droplets and their binary mixtures is presented to assess and address the motion, either attractive or repulsive, of both binary mixture droplets and pure droplets on a low pinning surface. A theoretical model based on the argument above is proposed to quantitatively analyze the interactions of pure or mixture fluid droplets. This finding is essential for understanding these phenomena and can shed light on exploiting this mechanism, such as design guidance for droplet-manipulation-based or microfluidic devices.

## Experimental Section

### Materials

Glass slides (AAAA000001##02E, Menzel Glaser) were purchased from Fisher Scientific (Product Code 12342108). HCl (1 M (mol/L), Sigma-Aldrich1099700001) and NaOH pellets (≥99%, Sigma-Aldrich1064980500) were used for surface cleaning. Propylene glycol (PG) stock solution (PG ≥ 99.5%, Sigma-AldrichW294004) was purchased for the use of pure PG and PG-water mixture preparation. Deionized water with a resistivity of 18.2 × 10^–6^ Ω·cm was acquired from a Barnstead NANOpure Diamond Analytical ultrapure water system. Polyethylene glycol (PG)/water binary mixture solutions with different concentrations were prepared by mixing the PG stock solution and DI water by weight.

### Preparation of Low Pinning Surfaces

Glass slides were sequentially sonicated in 2 M NaOH ethanolic solution (NaOH pellets dissolved in water and then mixed with ethanol) for 10 min, then in DI water for 5 min, in 1 M HCl for 10 min, and at last in DI water for 5 min. After cleaning, the surfaces were dried via the Marangoni drying method,^21^ where the water on the surfaces was driven away when exposed to the ethanol vapor, leaving behind a dry surface. The above surface cleaning procedure was the same as that described in Malinowski et al.^10^

### Preparation of the Pinning Location for the Droplet

Lines were drawn on the cleaned glasses with the ink of a marker pen (Staedtler Lumocolor) where the hydrophobic nature of the ink confines the droplets on the surface while at the same time creates pinning location for the “pinned” droplets. The ink was left to dry on the surface before the experiment to prevent any interference to the vapor profile.

### Experimental Procedure

During each experiment, a 0.5 μL “pinned” PG/water droplet and a 0.5 μL “free” PG/water free-moving droplet of given concentrations were deposited onto the cleaned surface within and outside the pinning location, respectively. A delay of 10 s was left between the deposition of the pinned droplet and that of the free-moving droplet to allow the development of a vapor profile around the pinned droplet. The behavior of the two droplets was recorded by a CCD camera with a framerate of 20 fps. An infrared camera was used to additionally capture the interactions from above. The experiments were conducted at a room temperature of 20 °C and humidity of 40% without the need for an enclosed chamber.

## Results and Discussion

### Phenomenon of Attractive and Repulsive Interactions

**Figure 1:**
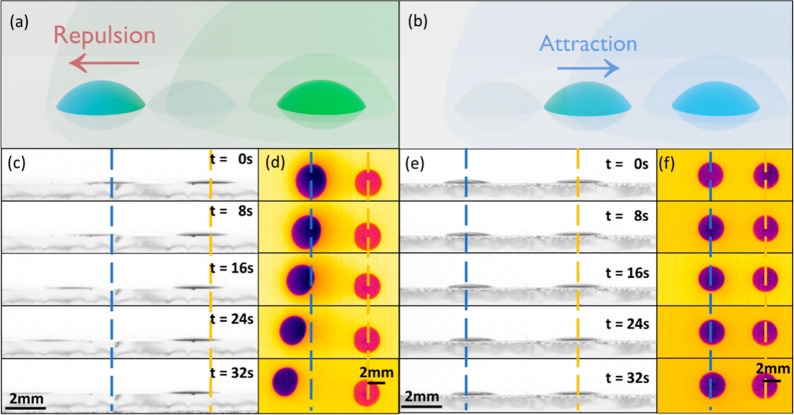
Attractive and repulsive droplet motion. Schematic diagram showing the (a) repulsive and (b) attractive interactions between a free-moving droplet and a pinned droplet. Both droplets could be initially pure or binary mixture droplets. Blue and green in the schematic diagram represent qualitatively the liquid components with higher and lower surface tension, respectively. The shadow near the left free-moving droplet indicates the previous location while the shadow around the pinned droplet indicates the vapor profile generated by it. Snapshots (c,e) are the side view optical images, and snapshots (d,f) are top view infrared images of the interactions between two droplets: (c,d) 0 vol % propylene glycol (PG) free-moving droplet and a 70 vol % PG pinned droplet and (e,f) 70 vol % PG of free-moving droplet and a 70 vol % PG pinned droplet. The free-moving droplet is on the left, while the pinned droplet is on the right. Blue and orange dashed lines show the initial center of mass of the free-moving and pinned droplets, respectively. It is noteworthy that the thermal pattern in the image does not represent the real temperature but the emissivity change with concentration. Evaporative cooling is negligible, as demonstrated in Section SI.2 in the Supporting Information.

We examine the interactions between two adjacent droplets: pure water, pure polyethylene glycol (PG) droplets, and/or their binary mixtures. In Figure 1, the interactions between a pinned 70 vol % PG droplet (right) and a free-moving pure water droplet (0% PG) or 70 vol % PG droplet (left) were shown. Under the same vapor profile of a pinned 70 vol % PG droplet, attractive and repulsive motion can be observed based on the concentration of the free-moving droplet.

The two cases presented in Figure 1 were chosen to show that both attractive and repulsive motion can be achieved under the same vapor profile from the pinned droplet (70% PG) by changing the concentration of the free-moving droplet (0% and 70% PG). This demonstrates that the attractive and repulsive motion can be achieved not only for a droplet with a greater difference in concentration, but also for similar concentrations. However, since the viscosity of the droplet increases with PG concentration, the attractive motion between two 70% PG droplets reported in Figure 1 is not as clear as the repulsive motion between 70% and 0% PG droplet.

### Complete Phase Diagram of Interactions

**Figure 2:**
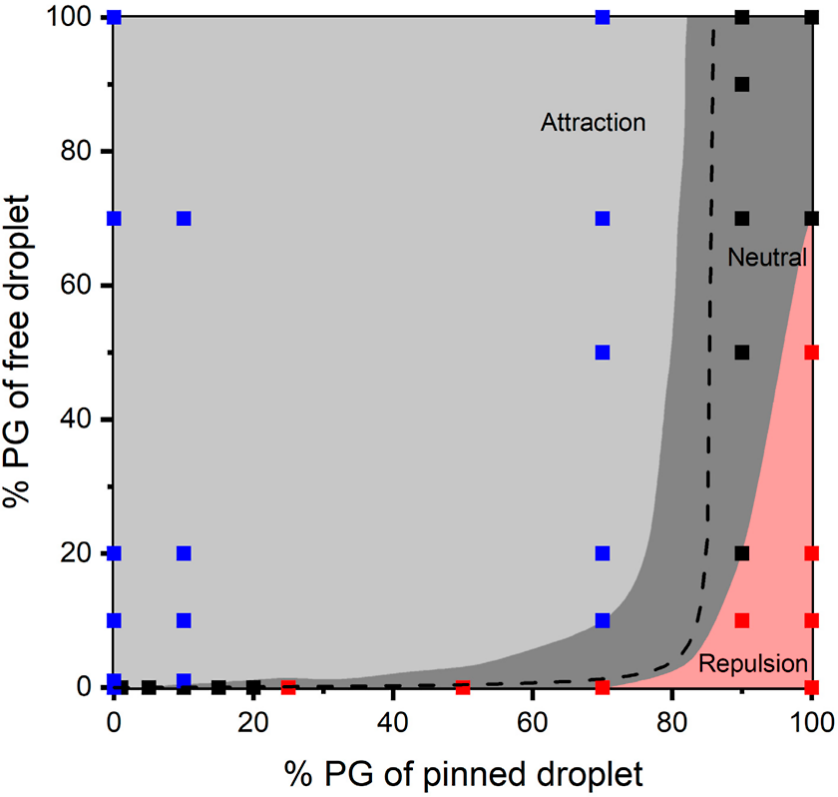
Complete phase diagram of contactless interactions between pinned and free-moving droplets. The volume of the droplets was 0.5 μL. Black dashed lines represent the theoretical critical concentration from eq 1. Comparison of the complete phase diagram with the literature can be found in the Supporting Information.

First, the droplet–droplet interactions as attraction, neutral, and repulsion are introduced in Figure 2 for water and for polyethylene glycol (PG) droplets and their binary mixtures accessing the complete range of pure and binary mixture concentrations (This is unlike in previous works: see Supporting Information Section SI.3 for comparison). A good qualitative agreement with the literature^14^ is reported for polyethylene glycol (PG) and water droplets with concentrations below 70%, which supports to some extent the importance of adsorption in adjacent droplet interactions and motion via the mediated vapor.^14^ In this study, a pinned droplet with 70% PG exhibited attractive interactions with both a free-moving droplet of 70% PG and a pure PG droplet. Conversely, the same pinned droplet demonstrated repulsive interactions with a free-moving droplet containing 0.1% PG as well as with a pure water droplet. The complete interaction phase diagram is found in Figure 2.

Figure 2 reveals that free-moving pure water droplets and pure PG droplets exhibit behavior similar to that of free-moving droplets in a PG-water mixture with very low and very high PG concentrations, respectively. This observation highlights the significant role of adsorption in the interactions between two adjacent droplets mediated by the vapor. Evaporation alone cannot account for variations in the surface tension of pure droplets (apart from the small effect of temperature change due to evaporative cooling: the droplet or substrate cooling effect due to the droplet evaporation^22^ is beyond the scope of this work). Besides, the different evaporation rate and the generated vapor gradient could only lead to repulsive motion due to the decreasing dependence of surface tension of water or PG on temperature.^14^ The complete phase diagram shown in Figure 2, including cases involving pure PG droplets, provides substantial evidence supporting the dominance of the adsorption mechanism.

**Figure 3:**
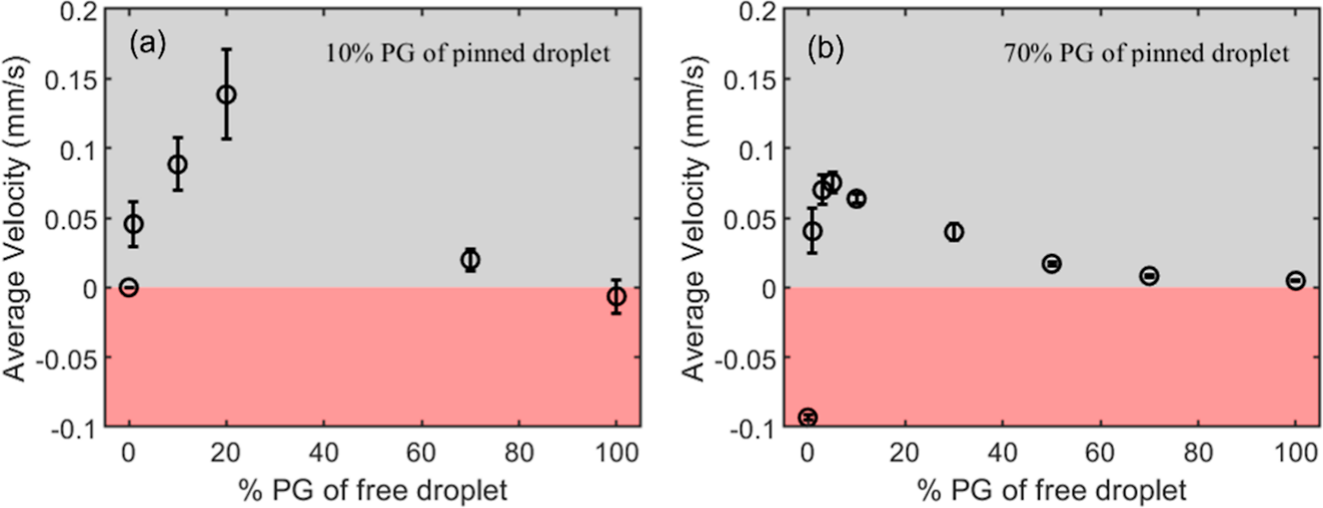
Average velocity of the free-moving droplet of different concentrations toward (a) 10 vol % PG and (b) 70 vol % PG pinned droplets. In shaded gray, the free droplet moving toward the pinned droplet exhibited a positive average velocity, i.e., attractive; while in shaded red, the free droplet moving away from the pinned droplet exhibited a negative average velocity, i.e., repulsion.

In addition, even when subjected to the same vapor profile, i.e., fixed pinned droplet concentration, the moving droplet experiences a different velocity based on its initial concentration, as illustrated in Figure 3.

For the case where the PG concentration of the pinned droplet is low, with the increase of initial PG concentration in the free-moving droplet, the average attractive velocity increases and then decreases, as represented in Figure 3a. For the case where the PG concentration in the pinned droplet is high, the repulsive motion is observed for the case of low PG concentration in the free-moving droplet, as shown in Figure 3b, and as represented in the bottom right corner of the phase diagram in Figure 2. We note here that if we consider the repulsive average velocity as negative and the attractive average velocity as positive, in both cases, the velocity increases with PG concentration to a maximum for low PG concentration and then decreases with the further increase of PG concentration in the free-moving droplet. This trend shows the transition of dominance of the different components in the vapor on droplet motion. At very low PG concentration in the free-moving droplet, the droplet is sensitive to the spatial distribution of the PG in the vapor. A higher level of PG vapor adsorption results in lower local surface tension, the gradient of which tends to move the droplet away from the vapor source, i.e., the pinned droplet. The component of water in the vapor contributes to the motion in the opposite direction as the existence of the water vapor gradient results in a lower water evaporation rate and a higher water adsorption rate at the front edge than at the rear edge with respect to the location of the vapor center. The overall mass transfer effect is shown as a gradient of water concentration along the droplet interface and therefore a surface tension gradient, which drives the moving droplet toward the pinned droplet. The direction of motion of the free-moving droplet is then determined by the superposition of the contributions of both water vapor and PG vapor, which depend not only on the vapor concentration but also on the initial concentration of the moving droplet itself. In addition, because both evaporation and adsorption take place simultaneously, our theoretical model and explanation are not restricted to the cases of mixture droplet pairs. They can also be applied to the interaction between pure droplet pairs or pure-mixture droplet pairs.

## Theoretical Model

In order to describe the moving direction and the dynamic motion of the droplet, a theoretical model is proposed based on both evaporation and adsorption enabling the accurate description, both qualitatively and quantitatively, of the vapor-mediated motion of the complete phase diagram presented in Figure 2, i.e., between droplets of mixtures, droplets of pure liquids, as well as mixture-pure droplet pairs. The main argument of the model is that during the interactions between two adjacent contactless droplets via surrounding vapor, whether binary or pure, both evaporation and adsorption processes take place for both components. The presence of the pinned droplet mediates the surrounding vapor profile, creating a vapor gradient for the free-moving droplet. The result of the mass transfer processes of the free-moving droplet with the surrounding vapor mediated by the pinned droplet leads to the concentration change within the liquid at the liquid–vapor interface of the microregion close to the triple phase contact line of the free-moving droplet. The spatial difference in concentration within the free-moving droplet results in the variation in local surface tension, which creates a net driving force along the vapor gradient and either attracts or repulses the free-moving droplet to the pinned droplet.

The magnitude of the mass transfer processes of both evaporation and adsorption and both components depends on the concentration of the two droplets and the (local) relative humidity. This theory is different from the mechanism proposed by Cira et al.,^14^ where only the evaporation rate of water was considered and the repulsive motion and/or the vapor-mediated motion for pure droplets could not be explained by this. In our model, the roles of adsorption and evaporation are regarded as equally important, and the significance of the PG vapor is considered as crucial as that of the water vapor.

In order to establish the relation between the initial concentration of the pinned droplet and the spatial vapor distribution, we introduce the water vapor pressure profile, *p*_W_, as a function of the distance, *r*, from the center of the pinned droplet, which is given by
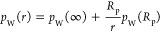
1

Equation 1 is a direct consequence of the radial diffusion equation^11^ with *R*_p_ as the contact radius of the pinned droplet. The water partial pressure at the contact line of the pinned droplet is *p*_W_(*R*_p_) = *p*_W_*α_p,W_ and the partial pressure in the ambient atmosphere is *p*_W_(∞) = ϕ*p*_W_*. Here, *p*_W_* is the saturated water vapor pressure at room temperature and ϕ is the relative humidity. While α_p,W_ is the water activity of the pinned droplet and equals the water mole fraction in the PG/water system,^23^*x*_p,W_ = 1 – *x*_p,PG_ (see Section SI.1 in Supporting Information), so the water partial pressure profile can be rewritten as
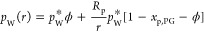
2For PG, the pinned or the moving droplet is the only source of PG, therefore, *p*_PG_(∞) = 0 and the vapor profile can thus be defined as
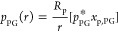
3where *p*_PG_* is the saturated PG vapor pressure.

**Figure 4:**
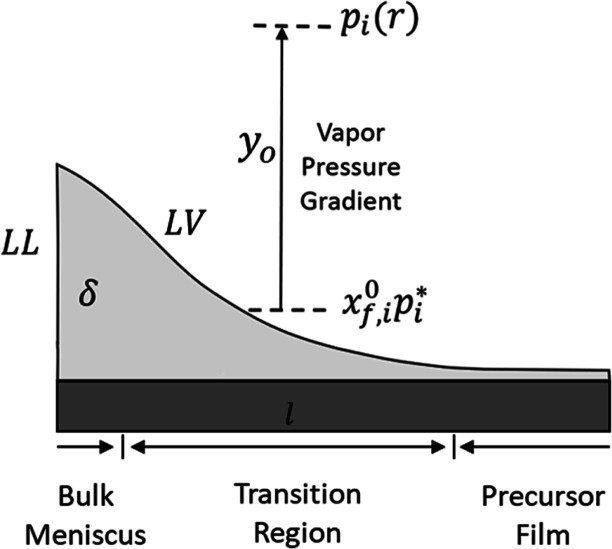
Schematic diagram of the microregion near the precursor film. δ is the maximum thickness of the microregion, and *l* is the microregion length before the precursor film. *y*_o_ is the distance from the local vapor profile generated by the pinned droplet, *p*_*i*_(*r*), to the vapor profile at the interface of liquid–vapor (*LV*), *x*_f,*i*_^0^*p*_i_*. *LL* is the boundary of bulk liquid and microregion.

The average mass change rate of PG and water through the liquid–vapor (LV) interface in the microregion can be then determined as^24^
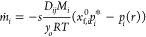
4where *s* is the area and equals *s* ≅ *l*·(unit width) for the microregion of a flat 2D droplet as represented in schematics in Figure 4. *D*_*ij*_ is the diffusion coefficient of the component *i* in the phase *j*, *M*_*i*_ is the molar mass of the component *i*, *R* is the ideal gas constant, and *T* is the room temperature. (*x*_f,*i*_^0^*p*_i_* – *p*_*i*_(*r*))/*y*_o_ is the vapor gradient of the component *i* above the microregion with a distance of *y*_o_. *x*_f,*i*_^0^ is the mole fraction of the component *i* in the microregion near the precursor film around the droplet.

As shown in Figure 4, the microregion is composed of three different sections, the bulk meniscus, where the mass transfer with the bulk droplet take place through the boundary LL, the transition region, where the mass transfer through the interface of liquid–vapor (LV) mainly takes place,^13,25^ and the connection with the precursor film. The height of the microregion is δ, which is of the order of several microns, and the height of the transition region is of the order of 0.1 μm,^25^ where the rate of mass transfer is maximal. Although the long-range van der Waals surface forces between the liquid and the solid could modify the precursor film composition,^26^ it can be assumed that the average mass concentration in the microregion, *c*_f,PG_^0^(0), is initially equal to *c*_f,PG_^0^(0) = *kc*_f,PG_, where *k* is a factor which depends on the droplet wetting properties for a given surface and relative humidity ϕ.

The molecules of both components of the liquid can transfer through the LV and LL interfaces. It is noteworthy that the mass magnitude of the bulk droplet is much greater than the microregion, and the mass transfer through LL interface will have little influence on the concentration of the bulk droplet. Here, we assume that the concentration in the droplet bulk can be taken as uniform due to relatively strong convection inside the droplet, at least during the early stage of droplet motion. Further, we apply the mass conservation in the microregion, and the fluid transferred through the LL interface is with the initial concentration of the microregion, *c*_f,PG_^0^(0).

Assuming the concentration of the microregion at time *t* to be *c*_f,PG_^0^(*t*), for an incremental time step Δ*t*, the mass transfer of PG through the LV interface and the LL interface equals  and , respectively. With mass conservation, the concentration of the microregion at the next time step (*t* + Δ*t*) is
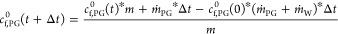
5where *m* is the mass of the microregion and *m* = *m*_PG_ + *m*_W_.

The fitting for the surface tension of the PG–water mixture on concentration is given by Chunxi et al.^27^
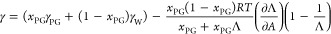
6where for PG and water, the parameter Λ = exp(−819.74/*T*) and ∂Λ/∂*A* = −423.16 × 10^–5^Λ/*T*, given by Chunxi et al.^27^

Before the droplet can move, it must overcome the pinning force at the contact line, *F*_p_. Therefore, the criterion for the droplet to move is *F* > *F*_p_ ∼ *R*(cos θ_rec_ – cos θ_adv_). Otherwise, we will have
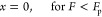
7which leads to the neutral region in Figure 2. For simplicity of the model, we assume *F*_p_ ≅ 0.

**Figure 5:**
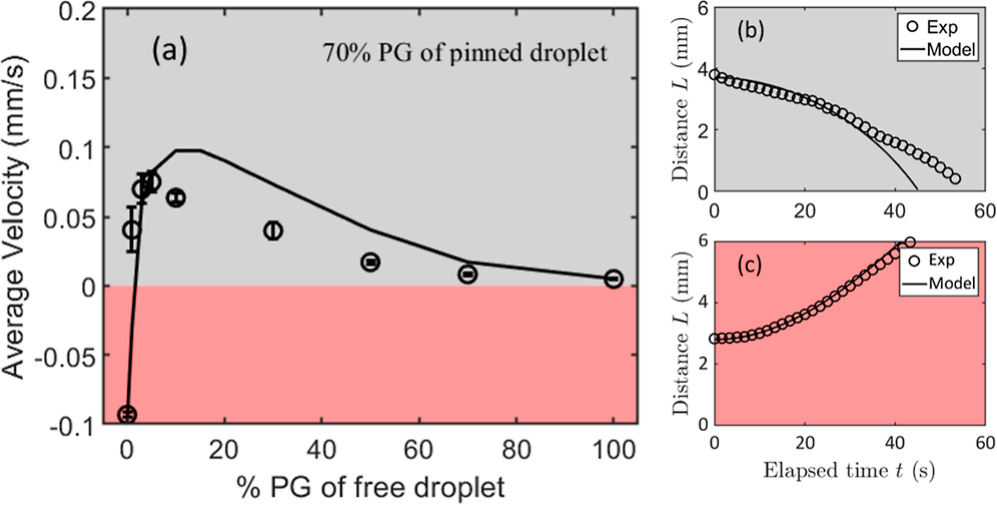
Comparison between experimental and theoretical model results. (a) Average velocity vs concentration of a free-moving 0.5 μL droplet moving toward a pinned 0.5 μL 70 vol % PG droplet. Edge-to-edge distance, *L*(mm), between a 70 vol % PG pinned droplet and a free-moving droplet of (b) 10 vol % PG during attraction and (c) 0 vol % PG (pure water) during repulsion.

Assuming the droplet is 2D (i.e., of constant section perpendicular to the direction of motion), . The surface tension difference between the front edge, γ_front_, and the rear edge, γ_rear_, of the droplet drives droplet motion, . This assumption with a thickness of  in the perpendicular direction to the motion leads to the same result as the case of a 3D spherical droplet under a gradient only in the direction of motion. With eqs 2–6, γ_front_ and γ_rear_ can be determined. We take the center-to-center distance between the two droplets to be *y*, and we assume a small contact angle in the microregion, θ_m_ ≪ 1, i.e., cos θ_m_ ≅ 1, which agrees with the experimental observation. We assume that the vapor gradient is only along the moving direction, the force acting at the droplet, *F*, eventually governing the dynamic motion of the free-moving droplet can be expressed as
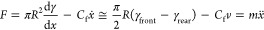
8where *C*_f_ is the drag coefficient,  and  are the velocity and acceleration of the droplet, respectively, *C*_f_ is a function of viscosity with *C*_f_ = 2.09 mN*s/m from the sliding experiment for a 5 vol % droplet on the same, tilted surface (ca. 3°), and the viscosity of the 5 vol % droplet is 0.8 mPa·s. Assuming the effect of the size of the droplet contact line is negligible due to the change of concentration,  in eq 8. With the viscosity, η, fitting from Khattab et al.,^28^ η = 21.8 × *x*_PG_^2^ + 17.1 × *x*_PG_ + 0.62 (mPa·s), *C*_f_ can be calculated. Assuming a maximal thickness of the microregion, δ, of 10 μm, the distance for the vapor gradient *y*_o_ = 3 mm and *k* = 1 for the microregion, integrating eqs 2–8, then the trajectory, i.e., position in time, of the free-moving droplet can be derived as represented in Figure 5b,c for the attractive and repulsive cases, respectively. The comparison of the average velocity between experiment and model is presented in Table 1. The model captured quantitively the trend from the experiments, and the average velocity derived from the model is of the same order of magnitude as that from the experiments. The deviation may come from the choice of values of the parameters mentioned above, such as the thickness of microregion, δ, the vapor gradient distance, *y*_o_, the initial concentration relation between bulk and microregion *k*, the form of friction coefficient, *C*_f_, the assumption of a spherical droplet, and the gradient only along the moving direction. A more refined adjustment could potentially enhance the alignment between the experimental and the model results. However, this would introduce additional complexity, which falls outside the scope of the present discussion.

**Table 1:** Comparison of Average Velocity between Experimental Measurement and Simulation for the Free-Moving Droplet with Varying Concentrations under the Vapor of a Given Pinned Droplet of 70% PG

initial concentration (vol %)	initial radius (mm)	calculated θ_0_ (deg)	υ_exp_ (mm/s)	υ_mod_ (mm/s)	|Δ| (mm/s)
0	1.75 ± 0.03	6.8 ± 0.3	–0.09 ± 0.00	–0.10	0.01
1	1.61 ± 0.05	8.7 ± 0.8	0.04 ± 0.02	–0.03	0.07
3	1.52 ± 0.06	10.3 ± 1.3	0.07 ± 0.10	0.06	0.01
5	1.55 ± 0.04	9.7 ± 0.7	0.08 ± 0.01	0.08	0.01
10	1.47 ± 0.02	11.4 ± 0.5	0.06 ± 0.00	0.10	0.03
30	1.38 ± 0.01	13.7 ± 0.3	0.04 ± 0.01	0.07	0.03
50	1.43 ± 0.02	12.4 ± 0.5	0.02 ± 0.00	0.04	0.02
70	1.46 ± 0.04	11.6 ± 0.9	0.01 ± 0.00	0.02	0.01
100	2.53 ± 0.13	2.3 ± 0.3	0.00 ± 0.00	0.00	0.00

Besides, the force acting on the droplet due to concentration difference (∼10^–7^ N) is 2 orders of magnitude larger than the force acting on the droplet due to evaporative cooling (∼10^–9^ N), therefore, the effect of evaporative cooling can be negligible. Details can be found in Supporting Information SI2. The model should predict the motion for both hydrophilic and hydrophobic surfaces as long as the pinning or contact angle hysteresis are low.

Figure 5a presents the comparison between the experimental results and those predicted by the model. In addition, the results of two examples of the model involve both attractive and repulsive interactions between the adjacent free-moving and pinned droplets, as shown in Figure 5b,c, respectively. The good qualitative and (to some extent) quantitative agreement between our experiments and the theoretical model proposed effectively captures the fundamental mechanisms underlying both attraction and repulsion. This supports our argument that the interactions between the two droplets arise from the combined effects of evaporation and adsorption.

### Critical Concentration

To investigate the direction of the motion as in the phase diagram in Figure 2, the change in surface tension due to concentration change at the microregion is compared between the “rear” and “front” edges of a freely moving droplet. Although the model assumes a simplified 2D droplet with a straight triple-phase contact line at both the front and rear edges, oriented perpendicular to the direction of motion, the fundamental physics remain consistent. From eq 5, the concentration change rate can be further expressed as
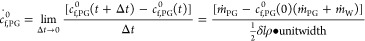
9

For the attraction, the surface tension in the microregion near the front edge of the droplet is higher than at the rear edge. Given that the surface tension of the mixture decreases with increasing PG concentration, the PG concentration is lower at the front edge than at the rear edge. Therefore, [*m*_PG_^·^ – *c*_f,PG_^0^(0)(*m*_PG_^·^ + *m*_W_^·^)]_front_ < [*m*_PG_^·^ – *c*_f,PG_^0^(0)(*m*_PG_^·^ + *m*_W_^·^)]_rear_. The repulsive motion leads to [*m*_PG_^·^ – *c*_f,PG_^0^(0)(*m*_PG_^·^ + *m*_W_^·^)]_front_ > [*m*_PG_^·^ – *c*_f,PG_^0^(0)(*m*_PG_^·^ + *m*_W_^·^)]_rear_. Assuming the distance between the two droplet mass center to be *d* = *L* + *R*_f_ + *R*_p_, *r*_rear_ = *d* + *R*_f_ and *r*_front_ = *d* – *R*_f_ with eqs 2, 3 and 5,  =  which leads to a critical concentration for the microregion being equal to  =  From eq 6, a transition from attraction to repulsion can be determined by a critical concentration of the free-moving droplet
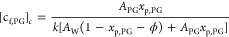
10where *A*_PG_ = *D*_PG_*M*_PG_*p*_PG_* and *A*_W_ = *D*_W_*M*_W_*p*_W_*, the products of the diffusion coefficient, molar mass and saturated vapor pressure of PG, *A*_PG_, and water, *A*_W_, respectively, being constant. In the model of Cira et al.^14^ (extended data Figure 5), droplets stay on the substrate until they reach to the equilibrium state, and the largest variation leads to *k* = 1.2. So, we assume *k* = 1 for simplicity in the model; this assumption is valid when the droplet is initially deposited onto the surface and the contact angle of the droplet is very small, θ ≪ 1. The critical concentration in eq 10 is based on the PG molar fraction of the pinned droplet, *x*_p,PG_, and the ambient relative humidity, ϕ.

**Figure 6:**
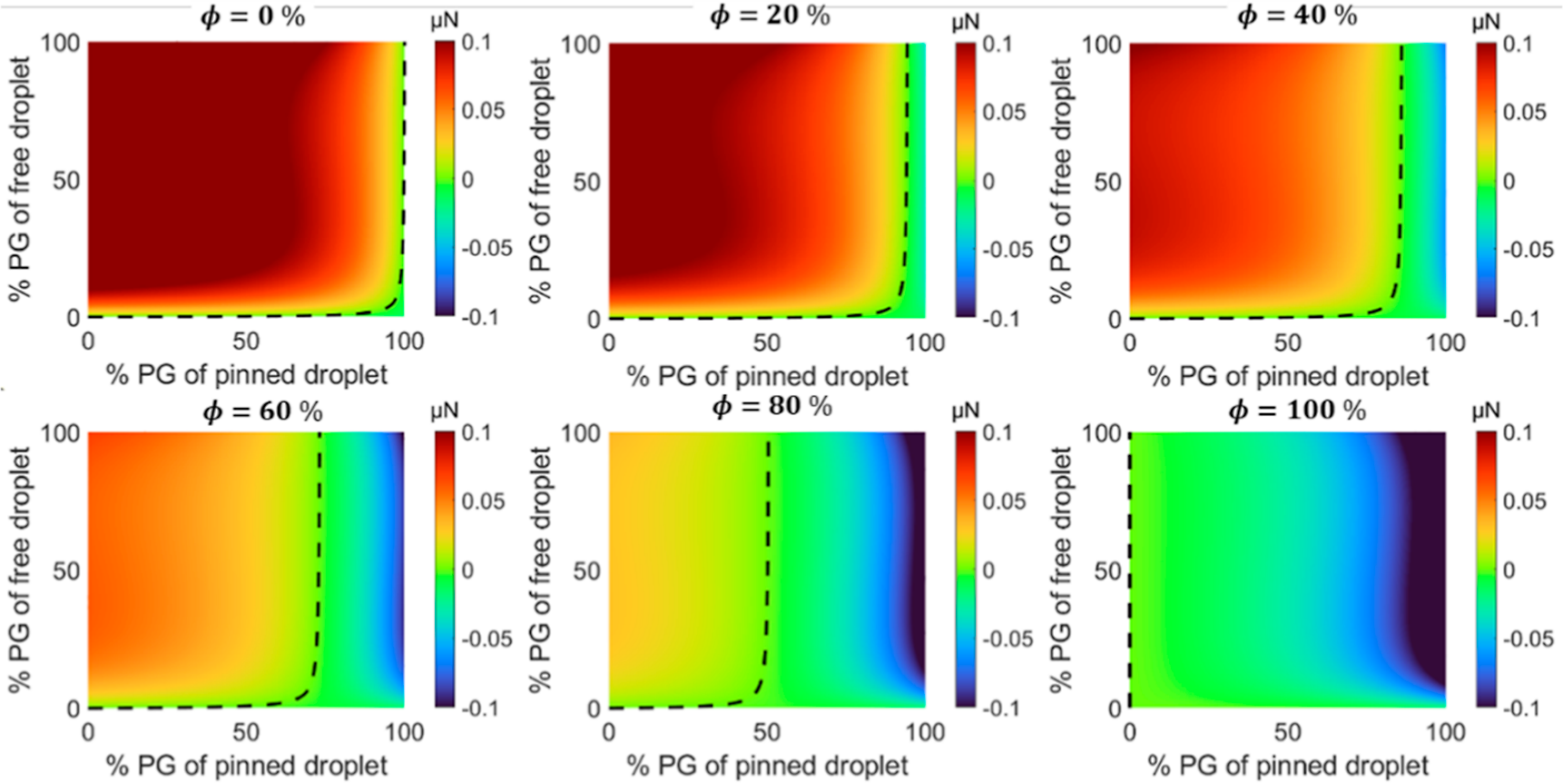
Force maps showing the dependence of the critical concentration on relative humidity, ϕ, with a fixed initial edge-to-edge distance, *L*. Force maps for a free-moving droplet before moving based on 1 s time step after deposition for mass transfer from the model proposed where the dashed line represents the critical concentration delimiting the attractive and repulsive regions. The edge-to-edge distance, *L*, between the two droplets is 4 mm.

**Figure 7:**
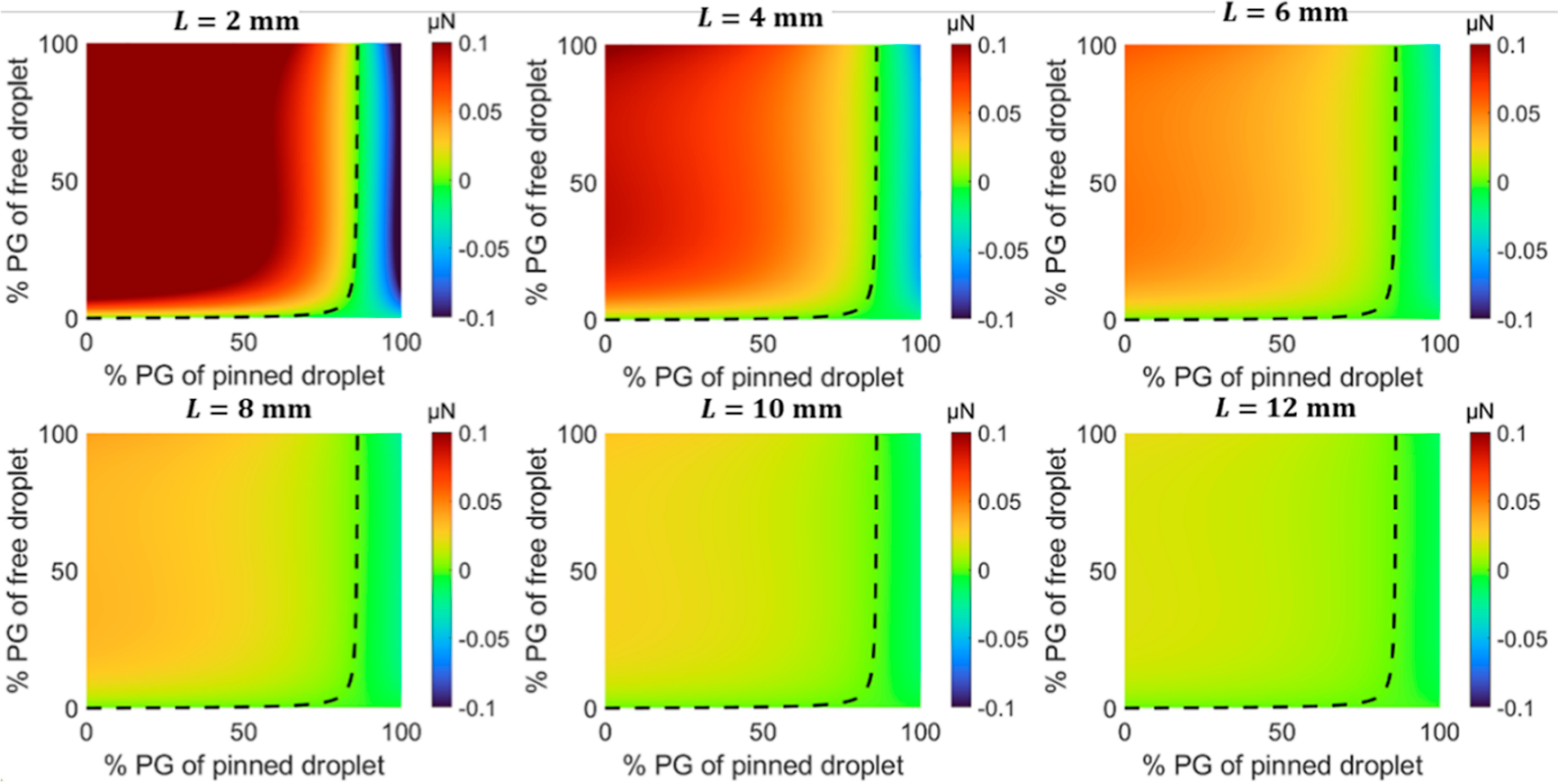
Force maps showing the dependence of the critical concentration on the different initial edge-to-edge distance, *L*, with a fixed relative humidity, ϕ. Force maps for a free-moving droplet before moving based on 1 s time step after deposition for mass transfer were shown from model. The relative humidity ϕ is 40%.

The black dashed line in Figure 2 represents the theoretical critical concentration, exhibiting a qualitative trend similar to that of the neutral boundary separating the attraction and repulsion regions. The mass concentration and mole fraction in the above critical concentration have been converted to the volume concentration in Figure 2. The area of the neutral region and the neutral cases in Figure 2 shall be caused by hysteresis. Besides, the dependences of the critical concentration on the concentration of pinned and free-moving droplets, relative humidity, ϕ, and distance, *L*, are shown in Figures 6 and 7.

Figure 6 presents the dependence of critical concentration on humidity and pinned droplet concentration, where the critical boundary shifts leftward with the increase in ϕ. It is noteworthy that to compare the results with Figure 2, the concentration is reported in volume rather than in mole fraction.

In addition, in Figure 7, we further demonstrate that the initial distance between two droplets only affects the magnitude of the driving force acting on the droplet and does not contribute to the critical concentration, at least within the limitations of the model. Consequently, the distance does not play a role in determining the direction of the interaction. Instead, three key parameters govern the direction of interaction between the droplets: the concentration of the pinned droplet, the concentration of the free-moving droplet, and the relative humidity.

Besides, the critical concentration, , has a limitation at 1-ϕ. For any pinned droplet with a PG concentration exceeding this limit, the free-moving droplet will be repelled from the pinned droplet, regardless of its own concentration. Specifically, when ϕ = 1, the free-moving droplet exhibits repulsive motion irrespective of the pinned droplet’s concentration. Within the concentration limit of the pinned droplet (*x*_p,PG_ < 1-ϕ), when the concentration of the free-moving droplet exceeds the critical concentration, the droplet exhibits attractive motion, whereas a concentration below the critical threshold results in repulsive motion of the droplet.

To have a better understanding of this limitation, eq 10 can be expressed in another way with the help of eqs 2 and 3, as
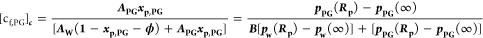
11where *B* = *D*_w_*M*_w_/*D*_PG_*M*_PG_ is a constant. This indicates that the critical concentration that determines the direction of droplet motion can be described as a function of the partial vapor pressure difference (or equivalently, the partial vapor concentration difference) of both PG and water between the pinned droplet and the surrounding environment.

## Conclusions

In summary, we have investigated the contactless interactions between two adjacent droplets for mixture droplets and for pure droplets on a low pinning surface both experimentally and theoretically. Attractive and repulsive motions have been observed for both pure and mixture droplets under the vapor profile generated by the adjacent fixed droplet. A mechanism considering solely evaporation could not explain either the motion of pure droplets nor the repulsive motion of both pure and mixture droplets; therefore, a theoretical model has been developed based on mass transfer coupling both evaporation and adsorption. The theoretical model successfully captures, explains, and quantitatively predicts the motion of both attractive and repulsive interactions for mixtures and pure droplets. Additionally, a critical concentration of the moving droplet was identified as the key factor separating the direction of the net interaction. This critical concentration is strongly influenced by the concentration of the pinned droplet and relative humidity. This work offers a more complete understanding of the vapor-driven motion of pure and mixture droplets relevant to surface science, engineering, and physical chemistry among others and with potential applications in fluid manipulation and microfluidics.

## Supporting Information Available

Additional discussion on the details of the work, including PG/Water activity, contribution of evaporative cooling, comparison with data in the literature, and data availability (PDF)
