# Supplementary material for: Long-Range Vapor-Mediated Interactions between Adjacent Droplets
Source: Langmuir. 2025 Feb 5;41(6):3986–94. doi: 10.1021/acs.langmuir.4c04255 (PMC11841030; doi:10.1021/acs.langmuir.4c04255)
Supplement: Supplementary file 1 — la4c04255_si_001.pdf [file la4c04255_si_001.pdf]

Supporting Information

Long-Range Vapour-Mediated Interactions between Adjacent Droplets

Hongyu Zhao[a], Daniel Orejon[a], Khellil Sefiane[a*], Martin E.R. Shanahan[b]

[a] Institute for Multiscale Thermofluids, School of Engineering, The University of Edinburgh, King's Building's, Mayfield Road, Edinburgh EH9 3FD, United Kingdom;

[b] University of Bordeaux, Arts et Metiers Paris Tech, I2M, UMR 5295, F-33400 Talence, France

[*]Corresponding Author: Khellil Sefiane. Email address: k.sefiane@ed.ac.uk.

Abstract

Droplet motion can occur due to interaction with the surrounding vapor phase. We examined experimentally the motion of two adjacent droplets, either pure liquid or a binary mixture, without direct contact. A droplet is repelled or attracted by the (pinned) adjacent droplet, which acts as a vapor source, depending on its initial concentration as well as the composition in the vapor, even for a pure liquid. The observation is explained by a theoretical model that combines evaporation and adsorption processes, which unifies the mechanism for both directions of motion (attraction and repulsion) and, more importantly, for both binary mixtures and pure liquid droplets. Good agreement is achieved between the theoretical model and the experimental observations. A critical concentration is proposed to determine the transition between attractive and repulsive motion, this being a criterion to predict droplet motion.

List of Content

SI.1 - PG/Water activity

SI.2 – Contribution of evaporative cooling

SI.3 – Comparison with data in the literature

SI.4 - Data availability

SI-5 – References

SI.1 - PG/Water activity

For a PG/water vapour system, the assumption that water obeys Raoult's law has been verified by Saxena and Hildemann[1]:

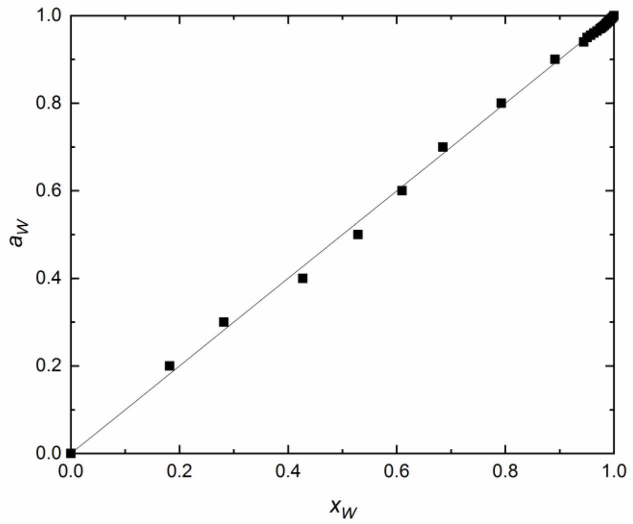

Figure S1 Water activity, a_W, *vs.* water mole fraction in the PG-water solution, x_W. Data points were redrawn in Figure 2a from Saxena and Hildemann [1]

Therefore, the water activity of the pinned droplet, $\alpha_{p,W}$, is equal to the water mole fraction in the PG/water system[1], $x_{p,W} = 1 - x_{p,PG}$. The PG-water system obeys Raoult's law, so the partial pressure for water and PG are $p_W = p_W^*(1 - x_{p,PG})$ and $p_{PG} = p_{PG}^* x_{p,PG}$ respectively.

SI.2 – Contribution of evaporative cooling

To calculate the contribution of evaporative cooling, assuming the only source of driving force is due to the evaporative cooling:

$$F_{cooling} \cong \pi R^2 \frac{dT}{dx}\left(\frac{d\gamma_{SV}}{dT} - \frac{d\gamma_{SD}}{dT} - \frac{d\gamma_{DV}}{dT}\cos\theta\right), \quad \text{(SI-10)}$$

where R is the radius of the droplet, γ_{SV}, γ_{SD} and γ_{DV} are the tensions at the solid-vapour, solid-droplet and droplet vapour interfaces. Given the fact that the droplet diameter is around 3mm, according to Hu & Larson[2], the temperature change for a similar sized droplet from apex to the contact line with around 10° contact angle at room temperature is around 0.005 °C, so dT/dx is around 3.3 °C/m. $d\gamma_{DV}/dT$ is the order of 0.17 mN/(m·°C), assume the maximum change of $\left(\frac{d\gamma_{SV}}{dT} - \frac{d\gamma_{SD}}{dT} - \frac{d\gamma_{DV}}{dT}\cos\theta\right)$ is with the same magnitude, $F_{cooling} \sim 4 * 10^{-9}$ N. Assuming one side of the droplet undergoes evaporative cooling and the other side does not, it leads to a driving force of $\sim 4 * 10^{-9}$ N due to cooling. Since the drag coefficient is measured to be $C_f = 2.09\ \mathrm{mN} * \mathrm{s/m}$ from the sliding experiment for a 5 vol.% droplet on the same, tilted surface (ca. 3°), and the velocity, v, is an order of 0.1 mm/s (Figure 5), the magnitude of driving force is $\frac{\pi}{2}R(\gamma_{front} - \gamma_{rear}) = C_f v \sim 1 * 10^{-7}$ N based on Equation 8, which also agree with the force map in Figure 6 & 7. therefore, driving force due to concentration changes is much larger than driving force due to cooling, $F \gg F_{cooling}$. Since PG is much less volatile than water, and the effect of cooling is overestimated in the analysis above, the effect of evaporative cooling for the pure water and PG droplets and their mixture is negligible.

SI.3 – Comparison with data in the literature

To support the idea that adsorption plays a fundamental role during adjacent droplet interactions and motion *via* the mediated vapour, we conducted experiments under similar conditions to those in Cira *et al.*[3]. Besides the qualitative agreement observed for similar conditions, we further extended the cases for pure water and for polyethylene glycol (PG) droplets with concentrations above 70% including the pure liquid as the freely moving droplet, which was not considered earlier. The comparison can be found in Figure S2.

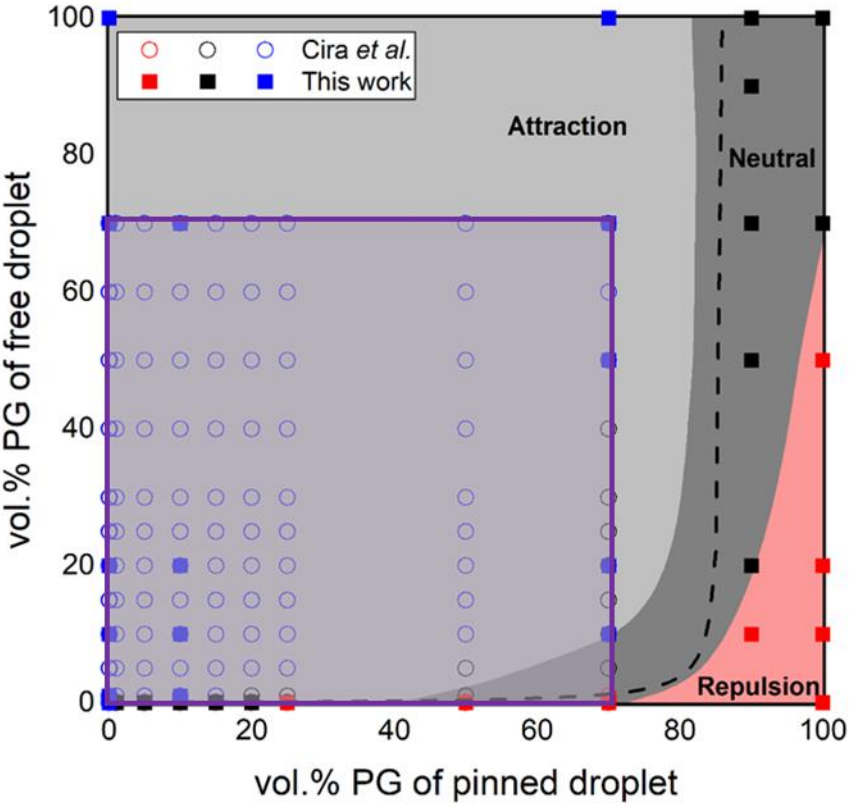

Figure S2 Comparison between our data and the data from Cira *et al.* [3]**.** The data from Cira *et al.* has been represented within the purple shaded area, which also includes our own experimental observations in solid symbols for direct comparison.

It is of interest that the boundaries of the three different regions in Figure S2 are not exactly the same as in Cira *et al.* Figure 3c. Subtle quantitative differences arise presumably due to the different surface treatments applied to our substrates, although the qualitative trend is quite clearly the same.

SI.4 - Data availability

The data supporting the findings of this study are available from the corresponding author on reasonable request.

SI-5 – References

1 Saxena, P. & Hildemann, L. M. Water absorption by organics: Survey of laboratory evidence and evaluation of UNIFAC for estimating water activity. *Environ. Sci. Technol.* **31**, 3318-3324 (1997).

2 Hu, H. & Larson, R. G. Analysis of the effects of Marangoni stresses on the microflow in an evaporating sessile droplet. *Langmuir* **21**, 3972-3980 (2005).

3 Cira, N. J., Benusiglio, A. & Prakash, M. Vapour-mediated sensing and motility in two-component droplets. *Nature* **519**, 446-450 (2015).
